# Bullous Pemphigoid Overlapping Psoriasis Vulgaris: A Rare Case Report and Literature Review

**DOI:** 10.3390/clinpract15050091

**Published:** 2025-05-08

**Authors:** Maria-Alexandra Timofte, Constantin Căruntu, Beatrice Bălăceanu-Gurău, Irina Mărgăritescu, Călin Giurcăneanu, Mara Mădălina Mihai

**Affiliations:** 1 Department of Oncologic Dermatology, “Elias” Emergency University Hospital, “Carol Davila” University of Medicine and Pharmacy, 020021 Bucharest, Romania; maria-alexandra.timofte@rez.umfcd.ro (M.-A.T.); calin.giurcaneanu@umfcd.ro (C.G.); mara.mihai@umfcd.ro (M.M.M.); 2 Department of Physiology, “Carol Davila” University of Medicine and Pharmacy, 050474 Bucharest, Romania; 3 Department of Dermatology, “Prof. N.C. Paulescu” National Institute of Diabetes, Nutrition and Metabolic Diseases, 011233 Bucharest, Romania; 4 Department of Pathology, Onco Team Diagnostic, 030138 Bucharest, Romania; irina.margaritescu@dermestetica.ro; 5 Dermestetica, 011854 Bucharest, Romania

**Keywords:** bullous pemphigoid, psoriasis vulgaris, hepatitis C virus, epitope spreading, genome-wide association studies, genotype–phenotype correlation

## Abstract

Bullous pemphigoid (BP) is a rare autoimmune disease, primarily affecting elderly individuals, that significantly impacts the patient’s quality of life. In contrast, psoriasis vulgaris (PV) is a common, chronic, immune-mediated skin condition recognized as a systemic T-cell-mediated disorder. We aim to present the case of a patient suffering from a dermatologic association of BP and PV, which unveiled hepatitis C viral infection as a potential trigger and led to complex therapeutic challenges. A literature review is also included, exploring previous cases of overlapping BP and PV, along with a discussion of the unique pathogenic mechanisms and an analysis of the available therapeutic options. The patient, a 53-year-old male with a seven-year history of PV, presented with tense bullae overlying the psoriatic papules and plaques, with a generalized distribution. The presence of hepatitis C infection was considered a potential trigger for the concurrent presentation of BP and PV. Recent GWASs have demonstrated a potential causal relationship between PV and the subsequent development of BP, suggesting shared genetic susceptibility and immune pathways. However, the exact mechanisms driving this transition remain incompletely understood. Our case is particularly relevant as it exemplifies how environmental triggers—such as chronic hepatitis C infection—together with chronic cutaneous inflammation may act as cofactors in this process, possibly through the ‘epitope spreading’ phenomenon. This case underlines the importance of identifying triggering factors in patients with overlapping autoimmune diseases and reinforces the need for future research to further elucidate the pathogenic link between genotype and phenotype, in order to improve personalized therapeutic strategies.

## 1. Introduction

The rare autoimmune blistering condition known as BP is caused by autoantibodies against the hemidesmosomal proteins of the skin and mucosal membranes: BP180 and BP230 [1]. Other target antigens such as Dystonin-e and collagen XVII are also involved [2].

BP affects most commonly elderly patients, with an increasing incidence over the past few years. Although the incidence of this disease varies between 2.4 and 23 cases per million each year, making it a rare condition, in those over 80, the incidence reaches over 312 cases per million [3]. On the other hand, BP is uncommon in people under 50, with stated incidence rates often lower—atypical for an autoimmune disease [3]. Studies that have looked into the predictive determinants of BP mortality demonstrated a link between aging and a poor outcome. Furthermore, overall health and low Karnofsky performance status have frequently been linked to higher mortality [4].

The pathogenesis involves an immune-mediated response, precipitated by medications such as antibiotics, psychotropic drugs, dipeptidyl peptidase-4 (DPP4) inhibitors, and diuretics; physical agents such as radiation, trauma, and ultraviolet (UV) exposure [1,3]; viral infections such as human immunodeficiency virus (HIV), hepatitis B virus (HBV), hepatitis C virus (HCV), Epstein–Barr virus (EBV), cytomegalovirus (CMV), and severe acute respiratory syndrome coronavirus 2 (SARS-CoV-2) [5], as well as vaccination against infectious agents, such as Neisseria meningitidis serogroup B, rabies virus, and SARS-CoV-2 [6,7]. Identification of trigger factors can increase the understanding of BP pathogenesis and easily improve the prognosis by removing the underlying cause.

The diagnosis of BP requires both clinical characteristics and laboratory tests. Direct and indirect immunofluorescence studies aid in diagnosis. Histology shows a superficial perivascular inflammatory infiltration, a subepidermal split, and a high concentration of eosinophils in the dermis [8]. In autoimmune blistering conditions, direct immunofluorescence is the gold standard of examination since it directly detects tissue-bound autoantibodies [8]. The direct immunofluorescence pattern for BP shows that C3 and immunoglobulin G (IgG) are deposited in the basement membrane zone in a linear, homogenous manner [8,9]. In the initial stages, C3 may be the only one identified [10]. The NC16A domain of BP180, commonly referred to as BPAG2, may be detected by Enzyme-Linked Immunosorbent Assay (ELISA) testing, with sensitivity ranging from 53% to 96% and specificity ranging from 94% to 100% [11]. In healthy individuals without BP, autoantibodies to BP180 and BP230 may be found, but they do not bind to the NC16A domain [8,12,13].

While PV is a chronic inflammatory disease, it was suggested that the changes in the basement membrane, with unmasked antigens, may induce an autoimmune response against BP antigens through cross reactivity and ‘epitope spreading’ and contribute to the development of BP [14]. In order to determine if PV makes it more likely to develop BP, Kridin et al. showed that, compared to the control group, patients with BP had a greater prevalence of pre-existing PV (1.7% compared to 1.1%) [15]. Moreover, BP may be induced by several therapies for PV such as phototherapy and biologic therapies [16].

We aim to present the case of a patient suffering from a rare dermatologic association of BP and PV, which unveiled hepatitis C viral infection as a potential trigger and led to complex therapeutic challenges. A brief literature review is also included, exploring previous cases of overlapping BP and PV, along with a discussion of the unique pathogenic mechanisms and an analysis of the available therapeutic options.

## 2. Bullous Pemphigoid Overlapping Psoriasis Vulgaris: A Rare Case Report

A 53-year-old male with a seven-year history of plaque-type PV was admitted to the Dermatology Clinic of Elias Emergency Hospital in Bucharest, presenting with newly developed blisters. In this case, the patient had no additional known comorbidities. He had never received systemic treatment or phototherapy for psoriasis and was not on any chronic medications known to be associated with BP at the time of presentation. This background supports the hypothesis of an endogenous trigger and contributes to the understanding of potential immune-mediated links between the two conditions.

**Figure 1:**
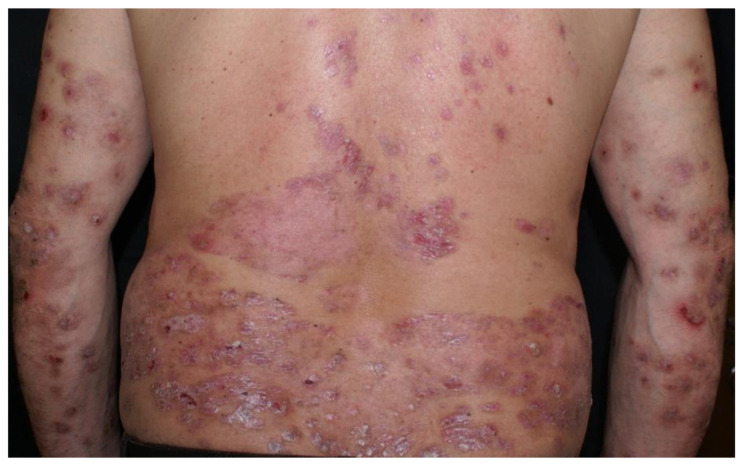
Psoriatic plaques and bullous pemphigoid bullae.

**Figure 2:**
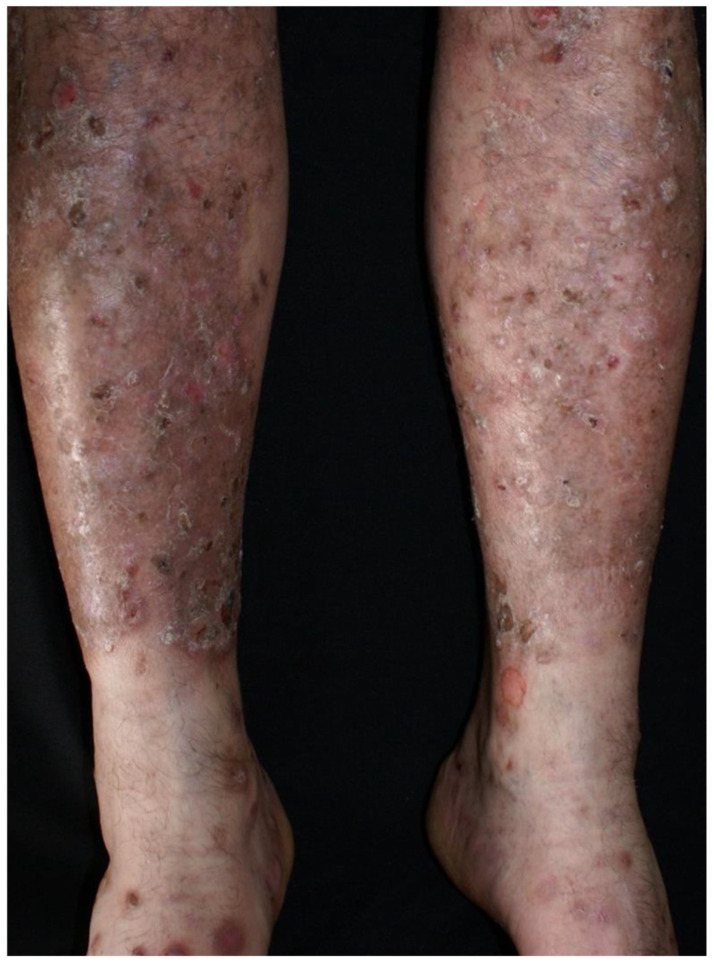
Psoriatic papules and plaques on lower legs, along with bullae.

**Figure 3:**
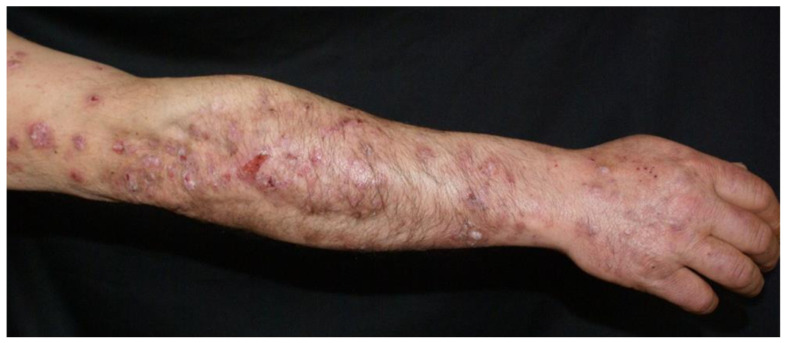
Erythemato-squamous papules and plaques along bullae on the upper limbs.

Clinical examination revealed a widespread eruption of thick, well-defined erythemato-squamous papules and plaques located on the scalp, extensor surfaces (elbows, knees, and lumbar area), and trunk (Figure 1, Figure 2 and Figure 3). Tense bullae with serocitrin content and an annular arrangement were observed, primarily overlying psoriatic lesions and, less frequently, on erythematous bases of perilesional skin in PV. An examination of the extremities showed nail pitting on both hands. No damage to the mucous membranes was detected, as there were no erosions or ulcers. The coexistence of PV and BP was suspected, prompting further laboratory tests and skin biopsies to confirm the diagnosis.

**Figure 4:**
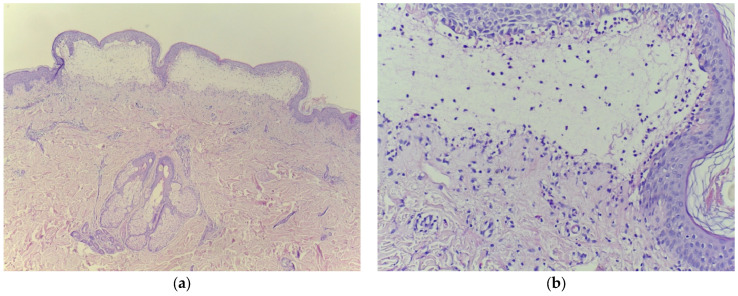
Histopathologic examination confirming bullous pemphigoid (**a**) subepidermal bubble, with fibrin, serum, lymphocytes, and eosinophils, HE 10×; (**b**) cleavage with erythrocytes is noticeable, HE 100×.

Laboratory tests showed a biological inflammatory syndrome with mildly elevated aspartate and alanine aminotransferase levels and positive testing for HCV. Tests for HBV, hepatitis D virus (HDV), and human immunodeficiency virus (HIV) were negative, while HCV RNA was detected using reverse transcription polymerase chain reaction (RT-PCR). Histopathological examination demonstrated sub-epidermal bullae and lymphocytic infiltration with eosinophils in the dermis, while direct immunofluorescence showed linear deposits of immunoglobulins (IgG, IgA) and C3 along the basement membrane, confirming BP (Figure 4).

While anti-p200 pemphigoid and epidermolysis bullosa acquisita (EBA) were considered in the differential diagnosis, our findings were more consistent with BP. Histopathology showed subepidermal blistering with eosinophilic infiltration, and direct immunofluorescence revealed linear IgG and C3 deposits along the basement membrane. Unfortunately, ELISA testing for BP180NC16a, BP230, and type VII collagen, as well as immunoblotting for 200 kDa and 290 kDa antigens, were not available in our center. However, the clinical, histological, and immunopathological features strongly supported the diagnosis of BP over other entities.

**Figure 5:**
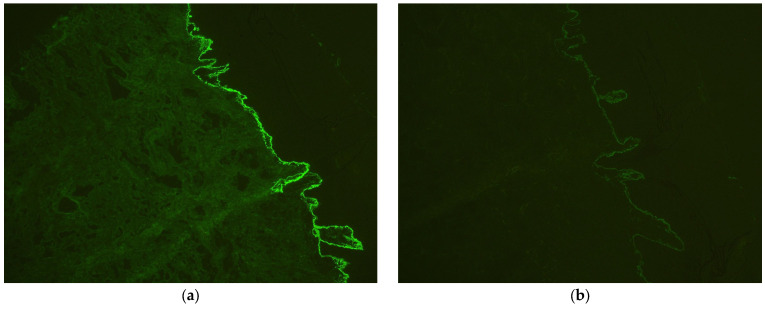
Direct immunofluorescence examination confirming bullous pemphigoid (**a**) linear deposits of immunoglobulins (IgG), 100×; (**b**) C3 along the basement membrane, 100×.

In cases of new-onset blistering eruptions in patients with PV, several autoimmune blistering diseases must be considered. EBA and anti-p200 pemphigoid can closely mimic BP both clinically and histologically [17]. EBA often presents with trauma-prone blisters, milia formation, and scarring, features that were absent in our patient [18]. Additionally, direct immunofluorescence typically reveals linear IgG deposition on the dermal side of salt-split skin in EBA, whereas in our case, direct immunofluorescence showed linear IgG and C3 deposits at the basement membrane (Figure 5) without access to salt-split indirect immunofluorescence [17]. Other conditions such as linear IgA disease, pemphigus vulgaris, and bullous drug eruptions were ruled out based on clinical morphology, histopathology, and the absence of relevant drug exposure. Given the combination of tense bullae, eosinophil-rich subepidermal blistering, and linear deposition of IgG/C3 on direct immunofluorescence, the findings were most consistent with a diagnosis of BP.

**Figure 6:**
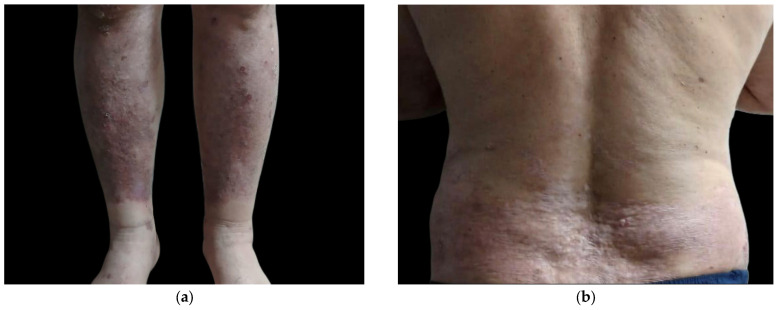
Follow-up clinical images demonstrating substantial improvement of bullous pemphigoid lesions following treatment. Lower limbs (**a**); posterior trunk (**b**).

The patient underwent a nine-week course of recombinant interferon alfa (INF-a) at a dose of 5 million units every other day. With close infectious monitoring, treatment included short-term systemic methotrexate (MTX) at 15 mg weekly which was used to control inflammation, folic acid supplementation, topical corticosteroid ointments, and psychological counseling. At a two-month follow-up, the patient exhibited a marked clinical response, with remission of bullous lesions and minimal erythematous desquamative lesions, reflecting sustained improvement (Figure 6).

## 3. Bullous Pemphigoid Overlapping Psoriasis Vulgaris: Potential Pathogenic Mechanisms

Recent studies suggest a pathogenic association between PV and BP mediated by chronic skin inflammation, T-cell-driven immune dysregulation, and the subsequent exposure of basement membrane antigens that may trigger autoantibody production [19]. Even if, among blistering diseases, BP is most frequently associated with PV, many other autoimmune bullous diseases were reported in the literature, including pemphigus foliaceus (PF), cicatricial pemphigoid (CP), linear bullous dermatoses (LAD), EBA (also named psoriasis bullosa acquisita), and pemphigus herpetiformis [20].

Shared immunogenetic and immunological mechanisms could be of significant relevance in the context of this disease association. PV has been linked to various other autoimmune disorders, including myasthenia gravis, Crohn’s disease, discoid and systemic lupus erythematosus, and ulcerative colitis [21]. It is plausible that psoriasis creates a specific immune system predisposition, which, under certain conditions, can trigger an autoimmune response [20,21]. The dysregulation of T-cell activity in PV might lead to the production of specific antibodies against basement membrane antigens [22]. One proposed mechanism is epitope spreading, where chronic cutaneous inflammation in psoriasis leads to the exposure of neoantigens, potentially initiating a secondary autoimmune response such as BP [23]. To better understand this coexistence, it is essential to investigate the immunopathological processes that may link these two conditions. Therefore, we will further examine the pathogenic mechanisms and triggering factors that may contribute to the development of BP in patients with pre-existing PV.

### 3.1. Genetic Factors

A recent study conducted by Wang et al., based on genome-wide association study (GWAS) data from multiple cohorts, provided important genetic evidence suggesting a causal relationship between PV and BP [24]. Their findings indicated that individuals with a genetic predisposition to psoriasis have a significantly increased risk of developing BP, while the reverse—BP predisposing to psoriasis—was not supported by the data. The absence of significant heterogeneity or pleiotropy in the analysis strengthens the validity of these conclusions. This study offers novel insights into the directional link between the two conditions and underscores the need for further research into the immunological mechanisms that mediate this relationship [25].

It is reasonable to consider that epigenetic alterations caused by PV lesions might initiate or enhance the autoimmune response to specific antigens, leading to the production of autoantibodies, the development of blisters, and a self-reinforcing cycle of organ-specific autoimmunity [26]. So far, there has been no evidence of a shared genetic link between BP and PV [27].

Autoimmune blistering disorders, including those classified as pemphigus and pemphigoid, are mediated by the immune system and are characterized by the presence of pathogenic autoantibodies in the circulation [28]. It is widely recognized that genetic factors significantly contribute to the development of these immune-mediated conditions and individual susceptibility. Recently, several human leukocyte antigen (HLA) genes have been linked to a higher risk of these diseases. Notably, HLA-Cw6 is crucial for antigen presentation and has been associated with PV [28]. This allele is among the most extensively researched in relation to PV susceptibility, with its prevalence in patients reported between 10.5% and 77.2% [28]. HLA-Cw6 not only increases the likelihood of developing PV but is also associated with a positive family history of the disease, an earlier onset of skin lesions, and a more severe clinical course. Additionally, this allele has been connected to guttate psoriasis, a higher occurrence of the Koebner phenomenon, and an improved response to treatments such as methotrexate and the anti-IL12/23 agent ustekinumab [28,29].

In a recent genetic cross-sectional study by Ciolfi et al., 40 patients with pemphigus vulgaris and 40 with BP were examined for HLA-Cw6 using the EUROArray test (Euroimmun Italia, Padua, Italy) [28]. The study found no statistically significant differences in HLA-Cw6 frequency among patients with pemphigus (*p* = 0.6368) or pemphigoid (*p* = 0.62) compared to a reference frequency of 0.086 [28]. Specifically, HLA-Cw6 was detected in 3 out of 40 pemphigus genotypes and 4 out of 40 pemphigoid genotypes. The frequencies in this cohort were 7.5% for pemphigus and 10% for pemphigoid, with no significant differences from reported prevalence in the Italian population [28]. None of the HLA-Cw6+ patients received methotrexate, limiting our ability to evaluate the predictive role of this allele regarding treatment response [28]. Additionally, PV patients were excluded from the study, which may have influenced the results [28].

### 3.2. Immunological Insights

PV has traditionally been classified as an immune-mediated Th1-type condition. Recent studies, however, have demonstrated that Th17 is the main pathogenetic subset of T cells and that it is also essential for autoimmunity [20]. Additionally, a key role in the pathophysiology is played by the proinflammatory cytokines interleukin (IL)-23 and tumor necrosis factor (TNF)-alpha [30]. A key factor in both PV and BP is interleukin (IL)-1, a cytokine that promotes inflammation and modulates the immune system [15]. It was found that IL-1 is necessary for the development and onset of psoriatic lesions and linked IL-1-regulated genes to signal transduction, proliferation, proteolysis, epidermal differentiation and adhesion [15]. According to another study, there is a correlation between IL-1β levels and illness severity [31]. Furthermore, in a pre-clinical animal model of BP, Il-1β has been demonstrated to enhance skin inflammation [15].

Chronic cutaneous inflammation in PV may contribute to BP development through a mechanism known as epitope spreading [32]. In this process, the persistent inflammatory environment leads to structural damage and disruption of the skin barrier, facilitating the exposure of previously hidden epidermal autoantigens, such as BP180 and BP230. Once exposed, these antigens may be recognized as foreign by the immune system, triggering a secondary autoimmune response directed against the basement membrane zone [32]. This transition from localized psoriatic inflammation to systemic autoimmunity underscores a potential immunopathogenic link between the two conditions [32]. The concept of epitope spreading thus provides a plausible explanation for why patients with chronic or uncontrolled psoriasis may develop BP, especially in the presence of genetic or environmental susceptibility.

Recent studies also suggest that gut microbiota alterations and oxidative stress may contribute to the systemic inflammation observed in psoriasis. A prospective study found significant correlations between microbiota composition, oxidative stress markers, and disease severity in treatment-naive psoriasis patients [33].

### 3.3. Potential Triggers

#### 3.3.1. Viral Infections as Triggers in Disease Development

While numerous predisposing and triggering factors have been reported, the contribution of viral hepatitis C to the development of BP remains unclear. Studies showed a notably higher prevalence of HCV, HBV, CMV, Helicobacter pylori, and Toxoplasma gondii antibodies in patients diagnosed with BP [34]. Furthermore, the COVID-19 pandemic has shed light on the potential effects of viral infections on autoimmune disorders. Although SARS-CoV-2 infection itself does not appear to significantly worsen the course of autoimmune bullous diseases, there are concerns about the triggering of new autoimmune conditions following vaccination [35]. The role of chronic infections and their possible synergistic effects with viral infections like SARS-CoV-2 underscores the need for ongoing research to better understand the immunopathological mechanisms involved in BP.

Studies have shown a significant difference between the prevalence of antibodies and that of different infectious agents, which is much higher in patients with BP compared to those not affected by BP [34]. In our case, the co-occurrence of BP and PV overlaid with HCV infection may support the theory that chronic infections contribute to autoimmune dysregulation through mechanisms such as molecular mimicry and chronic immune stimulation. Furthermore, the clinical presentation, with bullae forming on pre-existing psoriatic plaques, illustrates a potential real-life example of the ‘epitope spreading’ phenomenon.

Chronic HCV infection has been reported to be associated with numerous skin conditions. The most common are leukocytoclastic vasculitis and cryoglobulinemia. Furthermore, HCV infection has been linked to other dermatological conditions, such as porphyria cutanea tarda, lichen planus, erythema nodosum, urticaria, erythema nodosum, malacoplakia, pruritus, and erythema multiforme [36]. In individuals who are susceptible, HCV may act as a trigger for the onset of certain dermatological disorders. Patients should be checked for HCV until this notion is proven false, and those with active liver disease should think about receiving interferon treatment [36].

#### 3.3.2. Psoriasis Vulgaris Medications as Triggers in Disease Development

Individuals with PV may be receiving a wide range of treatment regimens, and any one of these agents may function as a trigger for BP [37]. Biologic-induced BP is a rare side effect of biologic treatments for psoriasis, typically associated with anti-TNF-α and anti-IL12/23 agents and rarely with guselkumab (anti-IL23) [38]. To date, drug-induced BP has been identified after psoriasis treatment with biologics including efalizumab, adalimumab, etanercept, secukinumab, guselkumab, and ustekinumab [38]. Previous studies have indicated that the mean latency period for biologic-induced bullous pemphigoid (BIBP) onset is approximately 5.12 ± 3.44 weeks with TNF-α blockers, compared to a longer duration of 28.66 ± 26.27 weeks for ustekinumab [16]. Notably, ustekinumab has been associated with the highest incidence of BIBP, particularly among patients who had prior treatment failures with TNF-α inhibitors [16]. Given the growing use of biologics in psoriasis, understanding the risk and characteristics of BIBP is essential for informed patient management.

Certain theories propose that phototherapy with ultraviolet B (UVB) or psoralen administration followed by UVA exposure (PUVA) modifies the antigenicity of the basement membrane, perhaps inducing an immune response likely to develop bullous disease [37].

#### 3.3.3. Non-Psoriasis Medications as Triggers in Disease Development

Recent studies have increasingly implicated a variety of medications as potential triggers for drug-induced BP, highlighting the need for heightened clinical awareness regarding these associations.

Non-steroidal anti-inflammatory drugs (NSAIDs), such as celecoxib and sulfasalazine, have been noted for their possible role in inducing BP through mechanisms involving hapten formation, although a significant association with aspirin was not observed in a United Kingdom case–control study [39,40].

Diuretics, especially loop diuretics like furosemide, thiazides, and aldosterone antagonists, have demonstrated a strong correlation with BP, with rechallenge studies confirming recurrence of lesions upon re-exposure [39,40,41,42,43].

In the realm of antihypertensives, angiotensin-converting enzyme (ACE) inhibitors, particularly those containing sulfhydryl groups like captopril, have been scrutinized for their potential link to BP, although results have been inconsistent across various studies [39,44].

Antibiotics, once thought to be rare contributors, have also emerged as potential triggers, with penicillins and quinolones being reported more frequently in BP patients [39,45,46].

Immune checkpoint inhibitors (ICIs), though effective in oncology, have been associated with de novo psoriasis or disease flares. Their immune-activating mechanisms may trigger psoriasiform reactions, complicating the therapeutic balance between oncologic efficacy and autoimmune tolerance [47].

Patients with PV are known to have a higher risk of developing BP compared to the general population, though cases involving the coexistence of these two conditions are rarely documented. A unique case of a patient who simultaneously developed generalized pustular psoriasis and BP was reported in the literature, nine months after the completion of immune checkpoint inhibitor therapy (pembrolizumab) for lung cancer [48]. Serologic testing revealed the presence of multiple autoantibodies (200 kDa proteins), laminin 332, and BP180 C-terminal [48]. Remission in both conditions was achieved through anti-interleukin 17 inhibitors for generalized pustular psoriasis and low-dose oral corticosteroids for pemphigoid [48]. While multiple autoantibodies are common in BP, detecting more than three distinct autoantibodies in one patient is unusual [48].

All these findings underscore the necessity for clinicians to maintain a high index of suspicion for BP in patients on these medications, facilitating early recognition and management of this autoimmune blistering disorder.

#### 3.3.4. Rare and Atypical Triggering Factors

Although uncommon, the existence of PV and BP has been reported in paraneoplastic contexts, particularly in association with glucagonoma, a rare neuroendocrine tumor of the pancreas. Several case reports have described either BP or psoriasis occurring in the setting of glucagonoma syndrome, suggesting that abnormal glucagon secretion and tumor-related immune dysregulation may play a role in triggering or exacerbating autoimmune skin disease [49].

In one case, a patient diagnosed with BP presented with necrotic and crusted cutaneous lesions and was later found to have metastatic glucagonoma, with histopathology confirming a pancreatic neuroendocrine tumor [49]. Another patient, initially misdiagnosed with pustular psoriasis, experienced multisystemic symptoms before glucagonoma was finally diagnosed via imaging and elevated glucagon levels [50].

These observations highlight the importance of considering underlying malignancies such as glucagonoma in patients with atypical, refractory, or multisystemic presentations of BP or psoriasis. Although rare, paraneoplastic processes may act as systemic triggers for cutaneous autoimmunity and warrant thorough evaluation in select clinical scenarios [51].

## 4. Bullous Pemphigoid Overlapping Psoriasis Vulgaris: Therapeutic Strategies

A more sophisticated approach to care is required when BP coexists with pre-existing PV, as this presents a significant therapeutic challenge. Therapeutic approaches that effectively address both disorders without aggravating one another must be carefully considered.

Autoimmune diseases frequently coexist due to common immunological pathways, requiring careful treatment strategies to address overlapping conditions without worsening either.

There is currently no accepted long-term, safe, and efficient treatment for patients who have both PV and BP. The severity of the condition, the patient’s tolerance, and the related pathologies are some of the factors that go into choosing the best course of treatment for these two autoimmune illnesses. A wide range of topical, systemic, and targeted therapies have been explored, either alone or in combination, depending on disease presentation. Table 1 provides an overview of therapeutic options reported in the literature for BP, PV, and their coexistence.

### 4.1. Topical Treatments

In the first instance, in less severe cases, topical treatments are the first choice, the most used being topical corticosteroids, in both BP and PV, but vitamin D3 analogs can also be an option in PV, or a combination of these treamtents [27]. Compared to oral corticosteroids, which are typically used as therapy for these individuals, strong topical corticosteroids are effective and have less adverse effects [37].

Beyond the topical treatments covered above, new therapeutic targets and strategies are being found for BP.

### 4.2. Phototherapy

More severe cases frequently require the initiation of systemic therapies and/or phototherapy with UVB or PUVA.

### 4.3. Immunosuppresive Systemic Therapy

Systemic corticosteroids and other immunosuppressants, such as methotrexate, mycophenolate mofetil, and cyclophosphamide, represent the primary treatment options for both BP and PV cases [27]. Methotrexate, as one of the most commonly prescribed systemic medications, has demonstrated a notable safety and efficacy profile in managing psoriasis associated with BP, both in our case and in those reported in the literature [27]. One reported case involved a male patient with both PV and BP that developed pustular psoriasis after systemic corticosteroid therapy [25].

### 4.4. Immune-Regulating Therapies

Other immune-regulating therapies that have shown potential efficacy in this context include dapsone, azathioprine, cyclosporine, a combination of erythromycin and etretinate, and others [52].

### 4.5. Biological Treatments

In psoriasis, biological therapies approved for treatment include anti-TNF-alpha, and anti-IL-12,- IL-23, and -IL-17 agents [30,53]. Several case reports and reviews have described the onset of BP as a paradoxical or adverse effect following these therapies, raising concerns about their use in patients predisposed to autoimmune blistering diseases [54].

Anti-TNF-α agents (e.g., adalimumab, etanercept, and infliximab) are widely used in PV and have been among the most commonly implicated biologics in BP induction. Cases of BP have been reported during or shortly after treatment with adalimumab and etanercept, with resolution upon discontinuation and initiation of immunosuppressive therapy [55]. Recently, a study reported a rare case of BP triggered by adalimumab in a patient with PV [56].

IL-12/23 inhibitors, such as ustekinumab, have also been associated with BP. Ustekinumab appears to have one of the highest incidences of biologic-induced BP among psoriasis treatments, particularly in patients who previously failed anti-TNF-α therapy [32].

IL-23 inhibitors, such as guselkumab, have rarely been associated with BP. One case described the development of BP after guselkumab therapy, though this appears less frequent compared to other classes [57].

Beyond its approved indication in plaque psoriasis, guselkumab, an IL-23p19 monoclonal antibody, has shown promising outcomes in complex autoimmune dermatologic scenarios. Notably, one reported case described successful treatment of anti-p200 pemphigoid in a patient with concomitant plaque psoriasis, suggesting that IL-23 inhibition may play a therapeutic role even in subepidermal autoimmune blistering diseases typically resistant to conventional immunosuppression [58]. In another challenging case, guselkumab led to sustained clinical remission in a patient with multirefractory BP, psoriasis, and psoriatic arthritis, where multiple previous lines of systemic and biologic therapies had failed [59]. These cases provide early but encouraging evidence that IL-23 inhibitors might serve as effective and well-tolerated options in patients with overlapping autoimmune conditions, including those with atypical or treatment-resistant forms of pemphigoid [59].

IL-17 inhibitors, including secukinumab and ixekizumab, though effective in psoriasis, have been linked to BP flares in patients with known or subclinical disease. Notably, some cases reported difficult-to-control relapses of BP after secukinumab initiation, suggesting a need for caution [60]. There have been reported two cases of patients with previously stable BP who experienced significant flares while on IL-17A inhibitors for PV—specifically, ixekizumab and secukinumab [61]. One patient, with pemphigoid induced by secukinumab, showed a particularly difficult-to-manage relapse [61]. These cases emphasize the importance of a thorough history of pemphigoid and BP180 autoantibody status in psoriasis patients before initiating IL-17A inhibitor therapy, urging caution among clinicians when considering these biologics for patients with a pemphigoid history [61].

Building on these observations, both secukinumab and ixekizumab have also been reported to improve BP lesions and blisters, particularly when used in combination with prednisolone, or even as monotherapy in patients with or without concomitant psoriasis [32,62]. However, ixekizumab has failed in a recent clinical trial. In contrast, new-onset BP has developed in some patients treated with these agents for unrelated conditions, further highlighting the complexity of their effects.

Taken together, these findings indicate that IL-17 inhibitors may exert both beneficial and paradoxical effects on BP, depending on the clinical context. This duality reinforces the need for further investigation to elucidate the mechanisms behind these outcomes [63].

Although secukinumab is generally well tolerated, only a limited number of cases have described the onset of BP following its administration. One proposed mechanism suggests that inhibition of IL-17A may lead to a compensatory skewing of the immune response toward a Th2 profile, potentially facilitating autoantibody formation against components of the basement membrane [64].

While such reactions remain rare, they emphasize the importance of vigilant clinical monitoring during biologic therapy and support the need for ongoing research into the dual role of IL-17 inhibitors as both therapeutic agents and potential triggers in predisposed individuals [65].

Okamura et al. (2025) reported the successful treatment of a patient with BP and PV overlap with bimekizumab, a humanized monoclonal antibody that inhibits both IL-17A and IL-17F [66].

Treatment with spesolimab, an anti-IL-36 antibody, led to complete remission of PV without aggravating BP, highlighting IL-36’s potential role in amplifying inflammatory responses in BP [67].

Interestingly, in certain situations, biologics like secukinumab or ustekinumab have been used successfully in patients with both BP and PV, either alone or in combination with immunosuppressive agents such as methotrexate, showing the complexity and context-dependent nature of their effects [16].

IL-17 inhibitors are contraindicated or used with caution in patients with concomitant inflammatory bowel disease (IBD), due to the risk of disease exacerbation. One case report highlights the importance of careful patient selection and close monitoring when managing individuals with both psoriasis and IBD [68].

Due to the potential of biologics to both induce and control BP depending on patient-specific factors, clinicians should perform thorough screening and consider prior history of autoimmune blistering disease before initiating these therapies.

### 4.6. Targeted Therapies

JAK inhibitors are gaining attention as targeted therapies that suppress Janus kinase activity, modulate the JAK/STAT signaling pathway, and inhibit key proinflammatory cytokine pathways. By doing so, they affect T-cell differentiation and help control cytokines involved in the onset of numerous inflammatory and autoimmune diseases [69].

Tofacitinib has been shown in the literature to be a safe and effective therapy for patients with psoriasis and BP together [70]. This has significant effects on how treatment plans are guided for both comorbid disorders. Additionally, a case study detailing the effective use of the JAK inhibitor baricitinib in the management of a patient with this association has been published [70].

Because it precisely targets JAK, upadacitinib, a selective JAK1 inhibitor, exhibits greater clinical potential than first-generation JAK inhibitors like baricitinib and tofacitinib [69]. This specificity not only improves disease management but also significantly lowers the risk of adverse reactions [69]. Case studies have demonstrated the effectiveness of upadacitinib in treating BP and nail psoriasis, and it is also approved for psoriatic arthritis management [69,71,72].

Upadacitinib, a highly selective second-generation JAK inhibitor, has shown potential in treating these complex cases. Observations in the literature indicate that upadacitinib is both promising and well tolerated, offering valuable guidance for effective treatment strategies in clinical settings [69]. The results indicate that increased Th17 cell activity and dysregulation of Th1 and Th2 functions may play critical roles in the development of both BP and psoriasis, suggesting potential therapeutic targets for treatment. Targeting the signaling pathways that regulate Th17, Th1, and Th2 cells could help slow disease progression and benefit patients with both conditions [69]. The JAK/STAT pathway is essential for controlling these T-helper cell activities, influencing the diseases’ onset and progression. JAK inhibitors, such as upadacitinib, can provide therapeutic effects by inhibiting this pathway [69].

### 4.7. Future Approaches

In refractory, unresponsive cases of BP, other options are represented by biological therapies such as rituximab (anti-CD20 monoclonal antibody) and dupilumab (monoclonal antibody that targets the IL-4 receptor) [30,53]. Other therapeutic approaches to investigate may include blocking immunoglobulin E (IgE) binding to mast cells, such as omalizumab (anti-IgE monoclonal antibody), and inhibiting eosinophils and eosinophil-derived IL-31, which may aggravate pruritus [73]. Along with other mediators associated with eosinophils, eotaxin and IL-5, which are also increased in the BP lesions, are appealing targets for therapeutic intervention.

### 4.8. Combined Therapies

Also, BP with coexisting psoriasis may be safely and effectively treated with secukinumab and methotrexate together [74].

Systemic corticosteroids, azathioprine, cyclosporine, and dapsone have all been reported as second line treatments [75]. Niacinamide, acitretin, and antibiotics with anti-inflammatory qualities have a limited impact [76].

The best way to treat BP and psoriasis together, however, is unknown [76].

**Table 1:** An overview of local and systemic treatments for PV and BP. “+” indicates that the referenced source supports the use of the listed medication for PV, BP, or both; “-” indicates the absence of such use in the referenced literature.

	Target	PV	BP	PV and BP (Case Reports)
**LOCAL TREATMENT**				
Calcineurin inhibitors	Calcineurin	+[60]	+[60]	-
Vitamin D3 analogs	Vitamin D receptor	+[60]	-	-
Corticosteroids	Glucocorticoid receptor	+[60]	+[53,60]	-
Phototherapy	DNA, T-cells	+[60,77,78,79]	-	-
PUVA	DNA, T-cells	+[60,79]	-	-
**SYSTEMIC TREATMENTS**				
**Conventional Systemic Therapies**				
Methotrexate	DHFR	+[60,77,78,79,80]	+[53,60]	+[55,75,81,82,83,84,85,86]
Cyclosporin	Calcineurin	+[60,77,78,79,80]	-	+[87]
Retinoids—acitretin	Retinoic acid receptors	+[60,77,78,79,80]	-	-
Corticosteroids	Glucocorticoid receptor	-	+[53,60]	-
Azathioprine	Purine synthesis	-	+[53]	-
Mycophenolate mofetil	Inosine monophosphate dehydrogenaze	-	+[53,60]	+[88]
Fumaric acids	NRF2 pathway	+[77,80]	-	+[22]
**Biologic Therapies**				
Anti- TNF-α				
Adalimumab	TNF-α	+[77,78,79,80]	-	-
Infliximab	TNF-α	+[77,78,79,80]	-	-
Certolizumab pegol	TNF-α	+[80]	-	-
Etanercept	TNF-α receptor fusion protein	+[77,78,79,80]	-	+[89,90,91,92]
Anti IL-12/IL-23				
Ustekinumab	IL-12 and IL-23, p40 subunit	+[77,78,79,80]	-	+[93]
Guselkumab	IL-23, p19 subunit	+[80]	-	-
Tildrakizumab	IL-23, p19 subunit	+[80]	-	-
Risankizumab	IL-23, p19 subunit	+[80]	-	-
Anti- IL-17				
Secukinumab	IL-17 receptor A	+[79,80]	-[64]	+[94,95]
Ixekizumab	IL-17 receptor A	+[79,80]	-	+[62,96]
Brodalumab	IL-17 receptor A	+[80]	-	-
Bimekizumab	IL-17 receptors A, F and AF			
Other Biologic Therapies				
Efalizumab	CD11a (LFA-1)	+[77]	-	-
Alefacept	CD2	+[77]	-	-
Omalizumab	IgE	-	+[53,60]	-
Rituximab	CD20	-	+[53,60]	-
Dupilumab	IL-4/IL-30	-	+[53]	-
Spesolimab	IL-36			
Targeted therapies				
Tofacitinib	JAK1/JAK3	-	-	+[70]
Baricitinib	JAK1/JAK2	-	-	+[70,97,98]
Upadacitinib	JAK1	?	?	+[69]
Apremilast	PDE4	+[78,79,80]	-	-
Deucravacitinib	TYK2	+[99]	-	-
**Others Systemic Therapies**				
Dapsone	DHFR	-	+[53,60]	+[22,85]
Doxycycline	30S ribosomal subunit	-	+[53]	-
				
Immunoglobulins	Broad immune modulation	-	+[53,60]	-
Immunoadsorption	Autoantibody removal	-	+[53]	-

BP—bullous pemphigoid; PV—psoriasis vulgaris; PDE4—phosphodiesterase-4; TYK2—tyrosine kinase 2; DNA—deoxyribonucleic acid; DHFR—dihydrofolate reductase; IL—interleukin; TNF-α—tumor necrosis factor alpha; MTX—methotrexate; AZA—azathioprine; MMF—mycophenolate mofetil; CsA—cyclosporine A; IFN—interferon; and JAK—Janus kinase.

## 5. Conclusions

In conclusion, the concurrent manifestation of PV and BP represents an intriguing scenario involving a complex immunological response and a wide range of precipitating factors that need to be identified. In this association of autoimmune diseases, patients should undergo assessments for infections such as HCV, which should be considered a precipitating factor that contributes to the exacerbation or onset of autoimmune blistering diseases.

The ‘epitope spreading’ hypothesis provides a plausible explanation for the coexistence of these two conditions. This case adds to the limited number of reports in which BP develops directly over psoriatic plaques and is associated with a chronic viral infection, supporting a potential pathogenic role of ‘epitope spreading’ and sustained immune activation.

Our article gains particular relevance in light of recent data from GWAS, which suggest a potential causal relationship between PV and BP. In this context, the identification of specific infectious triggers, such as hepatitis C virus in our case, or drug-related factors, including therapies commonly used in the treatment of PV (such as anti-TNF-alpha agents like adalimumab or anti-IL-17 biologics such as secukinumab and ixekizumab), which have been associated with the onset of BP, may provide a crucial link between phenotype and genotype, contributing to the understanding of the pathogenic mechanisms involved. Consequently, further studies are indispensable to advancing the understanding of the pathogenesis.

Driven by this perspective, we aimed to conduct a comprehensive literature review, bringing together current knowledge on the complex interplay between these two conditions, with the goal of generating new hypotheses and stimulating future research in this evolving field.

## 6. Future Directions

Given the complex interplay between PV and BP, future research should aim to clarify the immunogenetic pathways linking the two conditions. The absence of available or ongoing clinical trials investigating the association between PV and BP highlights an existing gap in current knowledge, leaving room for future research directions. Recent explorations into neuroimmune modulation have suggested a potential therapeutic role for botulinum toxin type A (BoNT-A) in localized, treatment-resistant psoriatic lesions, via its anti-inflammatory and neuromodulatory effects [100]. As biologic therapies become increasingly used in moderate-to-severe psoriasis, the importance of a multidisciplinary approach in managing patients with complex comorbidities—such as liver disease or viral hepatitis—has been emphasized in the recent literature. Periodic evaluation and collaboration between dermatologists and other specialists are crucial for safe, individualized treatment planning [101].

Larger cohort studies and prospective registries are needed to determine the true prevalence and risk factors for their coexistence. Moreover, further investigation into the role of infectious triggers, such as hepatitis C, and the paradoxical effects of biologic therapies may offer valuable insights into both prevention and personalized treatment strategies. Finally, translational studies exploring mechanisms such as epitope spreading could contribute to the development of targeted immunomodulatory interventions.
